# Alcohol and dental hygiene: the experience of Tuscan Alcohological Centre training on early identification and brief intervention methodology

**DOI:** 10.1186/1940-0640-8-S1-A62

**Published:** 2013-09-04

**Authors:** Sarah Santini, Chiara Camorali, Ambra Chiocchetti, Ilaria Londi, Tiziana Fanucchi, Gabriele Magri, Fiorella Alunni, Mariangela Spampinato, Valentino Patussi, Gianni Testino, Emanuele Scafato

**Affiliations:** 1 Italian Association of Dental Hygienists, Florence, Italy; 2 Tuscan Alcohological Center, University Hospital Careggi, Florence, Italy; 3 Alcohol Regional Center of Liguria, University Hospital San Martino, Genoa, Italy; 4 Center for Alcohol Research, National Health Institute, Rome, Italy

## 

The Tuscan Alcohological Center (CAR), since 2000, contributes to the prevention of alcohol-related problems by implementation of early identification and brief intervention (IPIB). In particular, since 2012, the CAR began to cooperate with the AIDI (Italian Association of Dental Hygienists [DH]) to promote dental hygienists courses in IPIB in Bologna and Florence. This paper presents the preliminary results of the first 2 training courses for dental hygienists initiated by CAR. In particular, we evaluated the effectiveness of the courses and the satisfaction of the participants. In addition, we present the first results of a research project designed to evaluate the effectiveness of AUDIT and brief intervention carried out during oral hygiene sessions at reducing alcohol consumption. The sample included 60 DHs from Florence and Bologna. The two training courses were evaluated by three questionnaires made “ad hoc” by the CAR. The data was analysed by the Statistical Package for Social Science (SPSS) statistical program. In particular we evaluated motivations and expectations and knowledge and expertise on alcohol before and after training. Moreover, we assessed the satisfaction of participants. Dental hygienists used IPIB in their work settings. The AUDIT questionnaire was used most often, with additional questions about lifestyle and the consumption of alcohol (grams) during the week. Prior to training, results showed DHs lacked alcohol knowledge; the majority did not know about brief intervention and didn't investigate alcohol consumption in their patients. At the end of the courses, participants showed a significant increase in knowledge and skills. The preliminary results of the research project show the effectiveness of brief intervention in this context: of 268 patients, a reduction in alcohol consumption was seen in 45% between the first and the second meeting.

